# Do Aspirin and Flavonoids Prevent Cancer through a Common Mechanism Involving Hydroxybenzoic Acids?—The Metabolite Hypothesis

**DOI:** 10.3390/molecules25092243

**Published:** 2020-05-10

**Authors:** Ranjini Sankaranarayanan, D. Ramesh Kumar, Janki Patel, G. Jayarama Bhat

**Affiliations:** 1 Department of Pharmaceutical Sciences and Translational Cancer Research Center, South Dakota State University, College of Pharmacy and Allied Health Professions, Brookings, SD 57007, USA; ranjini.sankaranarayanan@sdstate.edu (R.S.); janki.patel@jacks.sdstate.edu (J.P.); 2 Department of Entomology, University of Kentucky, Lexington, KY 40506, USA; rameshinsilico@gmail.com

**Keywords:** aspirin, flavonoids, cancer prevention, hydroxybenzoic acids, cell cycle, CDKs, colorectal cancer

## Abstract

Despite decades of research to elucidate the cancer preventive mechanisms of aspirin and flavonoids, a consensus has not been reached on their specific modes of action. This inability to accurately pinpoint the mechanism involved is due to the failure to differentiate the primary targets from its associated downstream responses. This review is written in the context of the recent findings on the potential pathways involved in the prevention of colorectal cancers (CRC) by aspirin and flavonoids. Recent reports have demonstrated that the aspirin metabolites 2,3-dihydroxybenzoic acid (2,3-DHBA), 2,5-dihydroxybenzoic acid (2,5-DHBA) and the flavonoid metabolites 2,4,6-trihydroxybenzoic acid (2,4,6-THBA), 3,4-dihydroxybenzoic acid (3,4-DHBA) and 3,4,5-trihydroxybenzoic acid (3,4,5-THBA) were effective in inhibiting cancer cell growth in vitro. Limited in vivo studies also provide evidence that some of these hydroxybenzoic acids (HBAs) inhibit tumor growth in animal models. This raises the possibility that a common pathway involving HBAs may be responsible for the observed cancer preventive actions of aspirin and flavonoids. Since substantial amounts of aspirin and flavonoids are left unabsorbed in the intestinal lumen upon oral consumption, they may be subjected to degradation by the host and bacterial enzymes, generating simpler phenolic acids contributing to the prevention of CRC. Interestingly, these HBAs are also abundantly present in fruits and vegetables. Therefore, we suggest that the HBAs produced through microbial degradation of aspirin and flavonoids or those consumed through the diet may be common mediators of CRC prevention.

## 1. Introduction

Cancer is a global disease, and more than 1 million cases of colorectal cancers (CRC) are diagnosed worldwide each year [1]. Due to the increasing prevalence of CRC in the recent years, there is an urgent need to develop effective strategies for its prevention. Efforts to discover chemo-preventive drugs have met with limited success although conventional drugs like aspirin have been shown to prevent CRC. In addition, an increasing body of evidence suggests that consumption of fruits/vegetables rich in phytochemicals can prevent the occurrences of CRC [2,3,4]. Interestingly, while aspirin is a widely used synthetic “drug”, it is primarily a compound derived from the naturally occurring salicylic acid that is abundantly present in plant sources [5]. Flavonoids are another class of phytochemicals found in plants, fruits and vegetables that are also linked to a decrease in the occurrence of cancers [6,7,8]. Following the intake of aspirin or flavonoids, they are subjected to metabolism, both in the gut and liver, and this process produces several metabolites, some of which are hydroxybenzoic acids (HBAs) [9,10,11,12]. The chemistry and pathways of HBA generation have been well characterized; however, their role in cancer prevention has not been extensively studied. In the recent years, there has been an increased interest to understand their targets and roles in cancer prevention. This review aims to highlight the potential role of HBAs, generated through aspirin and flavonoid metabolism, in CRC prevention.

In this review we provide a brief overview first on aspirin’s ability to prevent cancer, followed by a discussion on the known roles of flavonoids in cancer prevention. We then explain the pathways of aspirin and flavonoid degradation leading to the production of HBAs and the other sources of these compounds commonly found in the diet. In addition, we have also highlighted the in vitro and in vivo studies performed using HBAs, currently available in literature, demonstrating its chemo-preventive/therapeutic potential against numerous cancers. Finally, we propose the “metabolite hypothesis”, to explain the cancer preventive effects of aspirin and flavonoids through the generation of HBAs.

## 2. Aspirin and Cancer Prevention

Aspirin has become one of the largest selling pharmaceutical compounds in the world since its first clinical introduction by Bayer in 1899 [5]. In addition to its well-known analgesic, anti-pyretic and anti-inflammatory actions, it has many beneficial health effects including the reduced risk for cardiovascular disease and CRC upon regular consumption [13,14,15]. Aspirins efficacy to reduce CRC is reported to be between 20%–40%, and the evidence for this effect comes from multiple epidemiological and clinical studies which showed that its intake for 5 or more years reduces the risk associated with colorectal adenomas and carcinomas [16,17,18,19]. This is also supported by animal studies where aspirin has been shown to decrease chemically induced carcinogenesis in colorectal tissues [20,21]. These observations and evidences prompted the United States Preventive Services Task Force (USPSTF), to recommend “initiating low dose aspirin use for the primary prevention of cardiovascular disease and colorectal cancer in adults aged 50–59 years” in 2016 [22]. Additionally, in view of these compelling evidences, numerous clinical trials have been launched to address its efficacy against CRC [18,23,24]. Though aspirin has been recommended for the primary prevention of CRC by the USPSTF, its role in secondary and tertiary prevention has not been clearly established [25,26].

**Figure 1:**
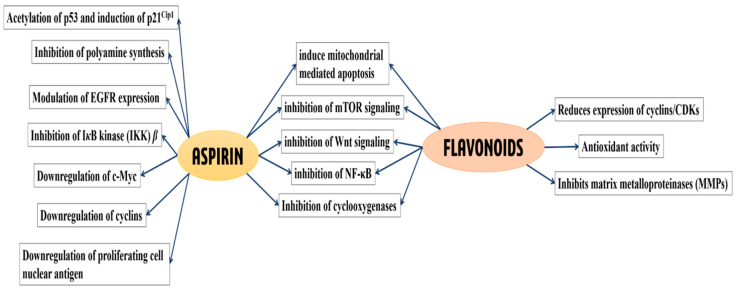
Classical pathways and cellular targets known to be affected by aspirin and flavonoids, leading to the prevention of various cancers. Aspirin and flavonoids affect numerous molecular pathways, some of which overlap. Pathways affected by aspirin alone are indicated in the left, shared pathways are shown in the middle, while pathways affected by flavonoids are shown in the right [6,13,14,33,34,35,36,37,38,39,40,41,42].

The intriguing aspect of aspirins ability to prevent CRC is that low doses (75–300 mg/day) are as effective as higher doses (≥500 mg/day) [27]. Interestingly, aspirin is also more effective against CRC when compared to cancers of the other tissues [28,29]. Numerous theories have been proposed to explain the potential pathways of cancer prevention by aspirin; however, a consensus has not been reached. The most widely discussed among them is the “platelet hypothesis” that implicates the inhibition of cyclooxygenase-1 (COX-1) enzymes in platelets as the contributing factor to cancer prevention [14]. As COX-2 overexpression is an important step in colon tumorigenesis [30,31] and as aspirin is more specific to COX-1 (IC_50_ 1.67 μM) than to COX-2 (IC_50_ 278 μM), the direct inhibition of COX-1 by low-dose aspirin is insufficient to explain its observed anti-cancer effects [18,32]. The platelet hypothesis hence proposes that aspirin’s chemopreventive effects may be attributed to the sequential inhibition of COX-1 and COX-2. The inhibition of COX-1 in platelets translates to prevention of both, platelet activation and release of cytokines/growth factors/lipid mediators at the site of gastrointestinal (GI) lesions, that eventually results in the inhibition of COX-2 expression in adjacent nucleated cells [14]. Though attractive, this hypothesis requires the orchestration of multiple events to exert the proposed preventive effects and is yet to be conclusively proven. Apart from the platelet hypothesis, other theories have been proposed including inhibition of mTOR signaling leading to the activation of AMP-kinase, inhibition of Wnt signaling, inhibition of NF-κB, inhibition of polyamine synthesis and modulation of EGFR expression, among others, and these have been reviewed extensively elsewhere [18,33,34,35,36]. A schematic of the pathways affected by aspirin is shown in Figure 1.

## 3. Flavonoids and Cancer Prevention

Epidemiological studies, short-term randomized controlled trials and preclinical studies in CRC patients have provided strong evidence in support of the cancer-preventive properties of flavonoids [2,6,7,43]. Flavonoids are subdivided into 6 categories based on their chemical structure—flavonols, flavan-3-ols, flavanones, flavones, anthocyanins and isoflavones [44]. These compounds are extremely sensitive to their environment and undergo rapid degradation in the presence of increased temperature and fluctuating pH. Additionally, they also degrade to simpler phenolic acids due to the actions of the gut microbes [44,45,46,47]. Most flavonoids are predicted to prevent the occurrence of cancer through various mechanisms that include their antioxidant properties, enzyme-receptor inhibition, regulation of apoptosis and the modification of signal transduction pathways which are described in several reviews. Interestingly, similar to aspirin, these compounds are also known to downregulate Akt/mTOR pathway, induce mitochondrial mediated apoptosis, inhibit NF-κB pathway, attenuate Wnt signaling, activate AMPK and suppress abnormal epithelial cell proliferation [6,37,38]. Despite these findings, it still remains unclear if the primary mediators of cancer prevention are the parent flavonoids or their degraded products. A schematic of the pathways affected by flavonoids is shown in Figure 1.

## 4. HBAs Are Generated through Aspirin and Flavonoid Metabolism

**Figure 2:**
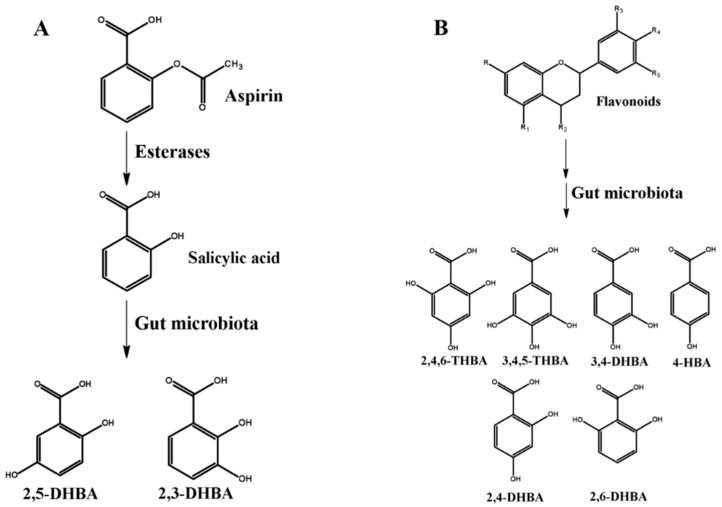
Metabolism of aspirin and flavonoids to generate hydroxybenzoic acids. (**A**) Aspirin metabolism generates 2,3-dihydroxybenzoic acid (2,3-DHBA) and 2,5-DHBA through CYP450 reactions in the liver [61]. DHBAs have also been shown to be generated through microbial metabolism of aspirin/salicylic acid [12]. (**B**) Flavonoid metabolism generates metabolites 2,4,6-trihydroxybenzoic acid (2,4,6-THBA), 3,4-DHBA, 3,4,5-THBA, 4-HBA, 2,4-DHBA, 2,6-DHBA through microbial degradation in the intestine [11,45,56,58]. R-R5 represent various functional groups (example -hydroxy, -ketone, -hydrogen, -methoxy, etc.) that are appended/attached to the flavonoid backbone to generate different groups of flavonoids.

The reported half-life of aspirin is about 20 min [48] and its metabolism either through cytochrome P450 (CYP450) catalyzed reactions [9] or direct conjugation of salicylic acid by phase 2 enzymes [10] in the liver has been well documented. Once absorbed, intact aspirin is partially hydrolyzed to salicylic acid (half-life is 4–6 h) by esterases in the blood and liver [49]. Salicylic acid can then be directly excreted (1%–31%) or can be metabolized in a number of different ways for elimination through kidney or bile. It undergoes conjugation with glycine to form salicyluric acid which accounts for 20%–65% of the metabolites generated, whereas ether and ester glucuronides of salicylic acid constitute 1%–42% of metabolites following conjugation with glucuronic acid [50]. Additionally, CYP450 enzymes in the liver can also metabolize salicylic acid to 2,5-dihydroxybenzoic acid (2,5-DHBA; gentisic acid) and 2,3-dihydroxybenzoic acid (2,3-DHBA; pyrocatechuic acid) that accounts for 1%–8% of the dose. 2,5-DHBA can further undergo conjugation with glycine to form gentisuric acid [9]. Aspirin has also been reported to be metabolized in the gut by the resident microflora. In this regard, Kim et al. demonstrated the importance of human fecal microbiota to degrade aspirin to salicylic acid and hydroxylated salicylic acids [12]. They showed that when rats were administered aspirin along with ampicillin, the bioavailability of aspirin increased when compared to rats administered with aspirin alone. Supporting this observation, a very recent study by Zhang et al., in 2019, also showed that administration of aspirin to rats following amoxicillin treatment decreased aspirin metabolism in the intestine, as compared to rats treated with aspirin alone [51]. The authors of both studies have suggested an important role for the intestinal microflora in the biotransformation of aspirin before its absorption into circulation. It is also important to emphasize that intestinal epithelial cells also express CYP450 enzymes [52], although their capability to generate these HBAs is not well studied. A schematic of the HBAs generated from aspirin is shown in Figure 2A.

The degradation of flavonoids, on the other hand, can occur through changes in physical parameters (like pH) [47], through microbial action in the gut before absorption [11,44], and through host metabolism in the liver [53]. It is reported that the absorption of flavonoids is 1%–15% in the intestine, and that it is extensively metabolized in the liver through conjugation reactions for subsequent elimination in the body [46,54,55]. It is also reported that these conjugated intermediates may be returned to the intestine through the bile, where it further undergoes deconjugation, subjecting them to further degradation through microbial metabolism [11,56]. The basic backbone of flavonoids is highly conserved and comprises of a benzene A-ring bound to a heterocyclic C-ring, which in turn is attached to a second benzene B-ring (Figure 2B). Depending upon the class of flavonoids, these rings are appended with different functional groups that confers their characteristic properties [57]. The functional groups, their number and position on this backbone will also determine their stability and the metabolite(s) they generate [58]. Flavonoids are generally stable under acidic conditions but undergo rapid degradation to simpler phenolic compounds under alkaline conditions (like in the intestine). Additionally, multiple studies have documented the ability of gut microbes to degrade flavonoids into simpler phenolic acids, many of which are HBAs. The most commonly observed HBAs include, 3,4-dihydroxybenzoic acid (3,4-DHBA; protocatechuic acid), 3,4,5-trihydroxybenzoic acid (3,4,5-THBA; gallic acid), 4-hydroxybenzoic acid (4-HBA), 2,6-dihydroxybenzoic acid (2,6-DHBA), 2,4-dihydroxybenzoic acid (2,4-DHBA) and 2,4,6-trihydroxybenzoic acid (2,4,6-THBA; phloroglucinol carboxylic acid) [11,44,46,47,55,59,60]. A schematic of the HBAs generated from flavonoids is shown in Figure 2B.

## 5. HBAs of Aspirin and Flavonoid Origin Exhibit Anti-Proliferative Effects in Cancer Cells

Studies carried out in our laboratory have demonstrated that aspirin metabolites 2,3-DHBA and 2,5-DHBA are capable of inhibiting Cyclin Dependent Kinase (CDK) enzyme activity and cancer cell growth, suggesting their potential role in CRC prevention [62,63]. Our study also demonstrated that 2,5-DHBA was effective in inhibiting cell proliferation in HCT-116 and HT-29 cells [63]. It is important to note that HCT-116 cells do not express COX-2 and HT-29 cells have inactive COX-2 [64], indicating that a COX-independent mechanism is at play. Supporting our observations, an in vivo study by Altinoz et al. also demonstrated enhanced survival in Ehrlich breast ascites carcinoma bearing mice upon oral administration of 2,5-DHBA [65]. Interestingly, other direct targets including FGF-receptors have also been identified for 2,5-DHBA [66].

Increasing evidences are now beginning to support the hypothesis that the degraded products are more likely responsible for the cancer preventive actions of flavonoids than the parent molecules [47,55]. A study by Peiffer et al. showed that administration of 3,4-DHBA to rats effectively inhibited NMBA-induced esophageal cancer [67]; in another study, it was demonstrated that 3,4,5-THBA inhibited prostate tumor growth and progression in TRAMP mice [68]. Several other reports have also documented that 3,4,5-THBA was effective in inducing apoptosis in a variety of cancer cells [59,69]. Experiments carried out in our laboratory have shown that 2,4,6-THBA inhibits cancer cell growth in cells expressing a functional monocarboxylic acid transporter (MCT) SLC5A8 [57].

Although in these studies effective inhibition of cancer cell growth by these HBAs required micromolar concentrations, it could be argued that the high levels of phenolic acid content observed in the gut arising from flavonoid rich food or other dietary sources is achievable [56,70], and may be sufficient to reach pharmacologically relevant concentrations to exert the observed inhibitory effect. Similarly, salicylic acid generated from the hydrolysis of aspirin may also reach micromolar concentrations in the gut as ~50% of orally administered aspirin is left unabsorbed in the GI lumen [71,72]. Hence, upon consumption of an 81 mg aspirin tablet, its concentration in the gut will be in the range of 0.3 mM to 1.4 mM under fed (~750 mL GI volume) and fasting (~160 mL GI volume) conditions, respectively [73]. It is important to note that not all HBAs are effective, as 4-HBA, 2,4-DHBA and 2,6-DHBA failed to inhibit cancer cell growth, suggesting that HBAs are selective in their modes of action [57,63]. It is also crucial to highlight the role of transporters in the uptake of these HBAs. In this regard, while the uptake of 2,4,6-THBA has been demonstrated to occur through the MCT SLC5A8 [57], transporter requirement for other HBAs has not been determined. In addition, while our studies have identified CDKs as potential direct targets for 2,4,6-THBA, 2,3-DHBA and 2,5-DHBA, the targets for 3,4-DHBA and 3,4,5-THBA are yet to be identified (Table 1).

## 6. Other Dietary Sources of HBAs

Published reports indicate that fruits and vegetables are rich sources of HBAs [74]. These HBAs produced as secondary metabolites in plants, majorly as byproducts of the shikimate pathway, have been implicated in plant defense against invading pathogens and also act as signaling molecules and antioxidants [3,74,75]. Salicylic acid, an HBA and the precursor for 2,3-DHBA and 2,5-DHBA, is widely found in many foods [76,77,78,79] and it has been argued that consumption of the spices rich in salicylic acid may account for low cancer incidence in rural India [77]. 2,3-DHBA and 2,5-DHBA are also reported to be present in dietary sources which may provide a direct link between the consumption of fruits and vegetable and reduced cancer risk. 2,3-DHBA is present in medicinal herbs such as Madagascar rosy periwinkle, *Boreava orientalis*, fermented soy products, in a number of fruits such as batoko plum, avocados and cranberries [75]. 2,5-DHBA is also found in abundance in plants and vegetables such as grapes, citrus fruits, *Hibiscus rosa-sinensis*, sesame, avocados, batoko plum, kiwi fruits, apple, bitter melon and black berries [75,80,81]. The flavonoid metabolite 3,4-DHBA is widely distributed in buckwheat, mustard, kiwi fruits, blackberries, strawberries, chokeberries and mangoes. Additionally, it is also present in chicory, olives, dates, grapes, cauliflowers and lentils. 3,4,5-THBA is reported to be present abundantly in tea, grapes, berries and chestnut [75]. All of these HBAs that have been demonstrated to be effective against cancer cell growth is also interestingly reported to be present in red wine along with 2,4,6-THBA [82]. These reports suggest that the presence of these HBAs of plant origin may have a positive effect on gut health that includes prevention of CRC.

## 7. The Metabolite Hypothesis—A Common Mechanism for Cancer Prevention

**Figure 3:**
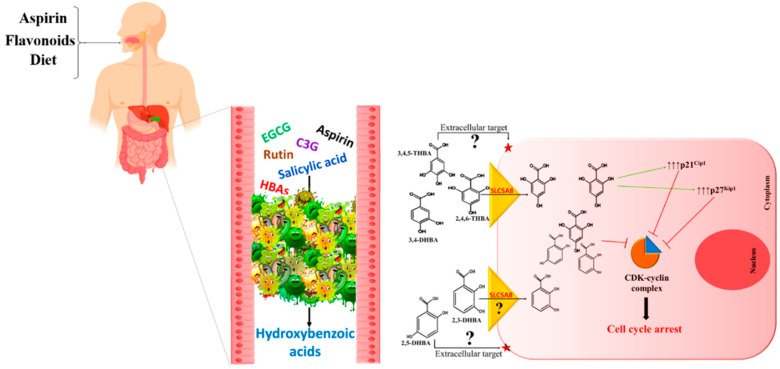
Metabolite hypothesis: Model depicting convergence of the pathways generating HBAs from parent compounds through host/microbial enzymes. We propose that actions of HBAs, generated through biotransformation of aspirin and flavonoids, retard rate of cell proliferation. This would provide an opportunity for (i) immune surveillance, leading to the destruction of cancer cells, or (ii) DNA repair in cells containing damaged DNA, providing genetic stability, both of which are important steps in the prevention of cancer. While 2,4,6-THBA is likely to retard cell proliferation through CDK inhibition and upregulation of p21^Cip1^ and p27^Kip1^, the exact mechanisms of cell growth inhibition by other HBAs is still not understood [57,63]. EGCG—Epigallocatechin gallate, C3G—Cyanidin-3-glucoside.

The unstable nature of flavonoids, the rapid hydrolysis of aspirin in the gut and the reports on the cancer-preventive potential of their degraded products through inhibition of cancer cell growth collectively suggest that the HBAs generated from flavonoids and aspirin may be key contributors to their cancer prevention properties. Although the parent compounds may directly contribute to the observed chemopreventive effects through other pathways already reported in literature (Figure 1), we suggest that the contribution of HBAs should also be taken into account. Unabsorbed flavonoids and aspirin/salicylic acid may act as substrates for the gut microbial enzymes to convert them into simpler, pharmacologically active HBAs, like 2,3-DHBA, 2,5-DHBA from aspirin and 3,4-DHBA, 3,4,5-THBA and 2,4,6-THBA from flavonoids. The generation of such HBAs in the gut compel us to propose a central role for these molecules in cancer prevention by aspirin, flavonoids and the diet. As these HBAs are metabolites generated through biotransformation in the body and are also secondary metabolites in plants with the capacity to inhibit cancer cell growth, we would like to refer to this hypothesis as “metabolite hypothesis”. A model depicting the convergence of the pathways generating HBAs from aspirin, flavonoids and diet through microbial/host enzymes is shown in Figure 3. The chemopreventive ability of aspirin and flavonoids have been established through many epidemiological studies and as the exact mechanisms of chemoprevention for these compounds have not been clearly established, we are suggesting through this review, that HBAs may be contributing to their chemopreventive actions. Though the data that was published previously on the ability of HBAs to prevent cancer cell growth were performed with cancerous cell lines, and therefore are more reflective of therapy than prevention, we believe that these observations can be extrapolated to cancer prevention. However, the extent to which HBAs are generated from these compounds and their overall contribution to the cancer prevention potential of the parent compounds require further investigation. Cancer prevention by HBAs is relatively underexplored, and further investigations are required to identify potential targets (intracellular vs. extracellular), their uptake mechanisms by cells, signaling pathways and target gene expression. Such studies would have tremendous implications in developing new strategies for cancer prevention.

## 8. Conclusions

It has been established over the course of time that increased consumption of fruits and vegetables has health benefits, making Hippocrates’ dictum “Let food be your medicine and medicine your food” all the more significant. Identification of polyphenols and phenolic acids commonly found as a component of the diet has now led to the generation of nutraceutical and/or pharmaceutical compounds that have revolutionized the health sector. Considering the increasing incidence of CRC worldwide, it is now more important than ever to come up with viable preventive strategies against CRC. We believe that HBAs merit further studies to understand their role in cancer prevention as the proposed mechanism (metabolite hypothesis) involving HBAs is a simple and tenable explanation for CRC prevention by both aspirin and flavonoids. Since intestinal epithelial cells are the first to get exposed to HBAs following their consumption or metabolism (from aspirin/flavonoids), in our view CRC prevention is likely to be a local effect. The colorectal tissues may thus have an anatomical advantage as they are the first to get exposed to these HBAs, making them preferred targets, while those HBAs absorbed into circulation may affect cancer development in other tissues as well. We believe that effective cancer prevention requires the partnership between the parent compounds and the right microbial species responsible for HBA generation. We also propose that the inter-individual variability previously observed in many epidemiological studies [8,17,27] could be attributed to the lack of an appropriate microbial ecology necessary for their degradation. Therefore, a strategy involving supplementation of the diet with appropriate probiotics and aspirin/flavonoids, or directly consuming HBAs may prove useful in CRC prevention.

## Figures and Tables

**Table 1:** Table showing the source of the hydroxybenzoic acid (HBA) metabolites, their potential to inhibit Cyclin Dependent Kinase (CDKs) and their ability to retard cancer cell growth.

Compound	Aspirin Metabolite	Flavonoid Metabolite	CDK Inhibition	Inhibition of Cancer Cell Growth	Reference
2,3-DHBA	+	-	+	+	[63]
2,5-DHBA	+	-	+	+	[63]
2,4,6-THBA	-	+	+	+ (in the presence of a functional SLC5A8)	[57]
3,4,5-THBA	-	+	-	+	[57,69]
3,4-DHBA	-	+	-	+	[57,67]
2,4-DHBA	-	+	-	-	[63]
2,6-DHBA	-	+	-	-	[63]
4-HBA	-	+	-	-	[57]

## Abbreviations

CRC	Colorectal cancers
COX	Cyclooxygenase
CDK	Cyclin dependent kinase
CYP450	Cytochrome P450
2,3-DHBA	2,3-dihydroxybenzoic acid
2,5-DHBA	2,5-dihydroxybenzoic acid
3,4-DHBA	3,4-dihydroxybenzoic acid
HBA	Hydroxybenzoic acid
IC50	Inhibitory Concentration -50%
MCT	Monocarboxylate transporter
3,4,5-THBA	3,4,5-trihydroxybenzoic acid
2,4,6-THBA	2,4,6-trihydroxybenzoic acid
FGF	Fibroblast growth factor
GI	Gastrointestinal
USPSTF	United States Preventive Services Task Force
